# Doctors, Ancient and Modern

**Published:** 1882-01

**Authors:** Ausburn Towner


### DOCTORS, ANCIENT AND MODERN.

AUSBURN TOWNER.

II.

More should have been added as to the origin of medicine and the early physicians than was said in my paper in the BISTOURY for October. As to its mythological origin, an allusion has already been made, and more will be said further on.

It seems a little singular that physicians receive so little respect in the pages of the Bible, for one of the highest functions performed by our Saviour when he was on earth was that of a physician or healer, and his ability in that direction was esteemed one proof of his divine origin. There was an idea indeed that diseases were strokes of divine displeasure or vengeance, and medicine was seldom used for their cure, what remedies there were being applied only in cases of outward sores, fractures, wounds or the like. Perhaps it was thought to be a sacrilege on the part of any man to attempt to cure a disease, this being God's province, and perhaps further, the present occasional claim that is made by some that they can cure disease by prayer has drifted down in its present modified form to us, as has many another superstition of the days of forty or fifty centuries ago.

In but one or two of the ten or a dozen times that physicians are referred to in the Bible are they named with anything but a half sneer. Jermiah, viewing the calamities of the Jews, cries out: "Is there no balm in Gilead? Is there no physician there?"[1] and St. Paul calls Luke the "beloved physician."[2] But in the first of these cases the term is used figuratively, and in the second only as a description, Luke being the chief point St. Paul meant to emphasize, and not the fact of his being a physician.

In Joseph's time, physicians embalmed the dead, for he commanded the physicians, his servants,[3] to embalm his father, Jacob, and they did it. These could hardly have been curers of diseases unless they were priests, and they could hardly have been priests, for they were Joseph's servants, and he would have had no such in his household. At the best, whatever they were, they did not occupy the honorable position held by physicians these days, who have rather to do with bodies before than after death. There was King Asa, too, son of Abijah, who reigned on the throne of Judah for forty-one years, about equal to the time of Victoria's reign now. He and his doctors had a severe rebuke. In the thirty-ninth year of his reign he was seized with that royal disease, the gout. "Yet in his disease," says the sacred writ, "he sought not to the Lord, but to the physicians."[4] His rebuke was, that after suffering two years he died.

Job, too, has a slap at the physicians. In the midst of his great suffering, and while reproving his friends, he cried out: "But ye are forgers of lies; ye are all physicians of no value;" as though such an expression struck the lowest key in depraved conduct.

1 Jer. viii: 22.

2 Col. iv: 14.

3 Gen. l: 2.

4 II Chr. xvi: 12.

In the New Testament, physicians fare no better. It was with a sneer that Jesus repeated the proverb, "Physician, heal thyself,"[5] and His expression: "They that be whole need not a physician, but the sick,"[6] was a mere figure of speech. When He was going with Jairus to cure that sick little daughter, too, it is related that "in the throng there was a woman who had suffered many things of many physicians, and had spent all that she had, and was nothing bettered, but rather grew worse."[7] No comment need be made on this, except that in the whole Bible, physicians do not make a very creditable showing.

Leaving these notions of physicians, we can go out into the world at large and see whence the probable origin of the science they practiced. Herodotus indicates what might readily have been the fact. He says that in Assyria and Egypt, when any one became diseased or fell sick, he was carried into some public place in the city. It was made the duty, either by law or custom, for each person who passed by to interrogate the sick one concerning his malady. This was done that some one of the inquirers might, perhaps, have been afflicted with a similar disease himself at some time, or had seen its operation upon another, and could communicate to the sick one the process by which his own recovery was effected, or by which, in any other instance, he knew the disease to be removed.

This method of the practice of medicine would hardly be practicable in these days, even if we had no doctors, for it must be in the experience of every one that remedies proposed by friends for certain diseases are as numerous as the friends themselves. I know of one man who was troubled with a simple but severe cold, who was recommended by his friends, one by one, to try his favorite remedies. Being rather amused at the number and variety of prescriptions offered, he kept count of them after an hour or two, and in the course of the day he was advised to try no less than seventy-two certain cures for his cold, no two of which were alike, and varying from catnip tea to a Russian bath! Another one had more than one hundred cures for his rheumatism presented, and in both cases the remedies were deemed infallible.

It is not unreasonable to suppose that the practice of medicine grew into something like a system, and Herodotus thinks it did, by this interchange of experience, persons who had been disordered in any part of their bodies writing down the names of the remedies from which they received benefit, and then others collecting them all together. It took a long, long time, by this method, to create a system or a science, but it was done.

One man in this modern age, in his own lifetime, which was not a very long one, discovered a principle and created a science or system, that from the success it has achieved and the position it has assured to itself in the world, seems to be more valuable and more correct than the notions that it took centuries and centuries to gather together and spread about. This man made no compilation from the experiments or experiences of others, but built up his theory and practice by experimenting on himself, his wife, and for aught I know his wife's relations, too. This was Hahnemann, who, to modern times, occupies a somewhat similar position to that held by Hippocrates, of whom I spoke in my last paper, in ancient times. There was this difference between them: Hippocrates gathered together in one mass all of the learning of his own and preceding times—no mean or simple task; he produced nothing new. Hahnemann, on the contrary, discarded all that had gone before him and presented to the world something new. It was not new in the economy of nature, but had never before been known to man. In the modern definition of terms, Hippocrates might be called a man of talent; Hahnemann a genius. And as a further illustration, Hippocrates might be likened to a man who makes a dictionary, a grand, great work, to be sure, when well done; while Hahneman might be likened to an author, who takes those same words, but so arranges them that they become immortal from the thoughts they express.

The imaginative and credulous ancients were not content with such an every-day and common place origin to the practice of the healing art as I have shown to have been likely. This is not singular. Believing as they did, that disease was an evidence of Divine wrath, and seeing some beloved one who was stricken down and ailing, recovering at the hands of some wise man, how easy to assume that that man must be allied to the gods, if not himself a god, carrying as he did, or appeared to them to do, the power of life and health. Is it an uncommon thing in this practical age for parents to appeal to their

5 Luke iv: 23.
6 Mat. ix: 12; Mark ii: 17; Luke v: 31.
7 Mark v: 26; Luke viii: 43.

well-loved doctor to save a child from death, or after he has rescued him from the jaws of the grave, bless and cover him with favors as though he himself had a divine power? Our own consciousness will suggest to us how much easier it is to believe that Esculapius once lived, a man, rather than that he was a god, or a myth typifying the sun,[8] the first of which the Greeks made him, and the second of which modern investigators would reduce him to.

The manner in which the classical nations manufactured their mythology is not a very creditable one, and excites the wish that the expression of "Bob" Ingersoll: "An honest god is the noblest work of man," might the rather have been the principle that guided them. They had a way of transporting their eminent men direct to heaven after they died, and giving them a seat along with their other gods. To entitle them to this distinction, however, it was necessary that they should have some god's or goddess's blood in their veins. The proper arrangement of this, it will easily be seen, was not calculated to keep up the good name of the father or mother of the person to be deified. The people did not stop at this, however. Thus Esculapius was said to have been, not the offspring of an honest father and mother, but the child of the god Apollo and the nymph Coronis. It can never be known what the father and mother thought of this, and, indeed, it was not made known until after they died, but we have no doubt they cared for him as if he had been their own child. Certain it is, as shown from his subsequent life, that they gave him a good education.

Some curious stories are told of him that are not without interest. Before he was born the raven was a white bird! It must have been a croaker, however, just as it is now, for it went to Apollo with a long story of some very improper conduct of which Coronis had been guilty. Apollo was very angry, so angry that he first changed the snowy plumage of the bird to a dead black, as it has since remained, for its officiousness, then he removed Esculapius from Coronis and put her to death. It is said of Esculapius also, that he went with the hero Jason to Colchis, and that he accompanied the Argonauts as surgeon-in-chief of the fleet when they went after the golden fleece; that Minerva gave to him some of the blood from the veins of that awful creature, Medusa, with part of which he could destroy life, and with another part he could heal the sick, and that he brought back to life certain dead men.

We are all well aware that these are all idle tales, but they show that human nature was much the same forty centuries ago as it is to-day, and that there is no subject on which men and women are more gullible than that relating to their health. The stories told not only of the eminent man I have named, but of others engaged in the same line of business in those old days, sound very like in character to some of the medical advertisements in our newspapers, with this difference, however, that with all the exaggeration in the case of those, the fact remains of the existence of great and useful men, while in these there is nothing at all behind the exaggeration.

8 Esculapius, or as some scholars call him, Æsculapius, or as the latter pedants have him, Asklepios, under which latter term few would know who is meant, is a prominent example of how far back we go in our language for terms to describe common notions amongst us. Of course the adjective esculapian, applying to matters of health, is derived from his name, and hygiene or hygienics, a still commoner term for the science which treats of the preservation of health, and panacea, another common word, spring from the names of his two daughters, Hygeia and Panacea.

